# Sex hormones have pervasive effects on thymic epithelial cells

**DOI:** 10.1038/srep12895

**Published:** 2015-08-07

**Authors:** Maude Dumont-Lagacé, Charles St-Pierre, Claude Perreault

**Affiliations:** 1 Institute for Research in Immunology and Cancer, Université de Montréal, Montreal, QC, Canada H3C 3J7; 2 Department of Medicine, Université de Montréal, Montreal, QC, Canada H3C 3J7

## Abstract

The goal of our study was to evaluate at the systems-level, the effect of sex hormones on thymic epithelial cells (TECs). To this end, we sequenced the transcriptome of cortical and medullary TECs (cTECs and mTECs) from three groups of 6 month-old mice: males, females and males castrated at four weeks of age. In parallel, we analyzed variations in the size of TEC subsets in those three groups between 1 and 12 months of age. We report that sex hormones have pervasive effects on the transcriptome of TECs. These effects were exquisitely TEC-subset specific. Sexual dimorphism was particularly conspicuous in cTECs. Male cTECs displayed low proliferation rates that correlated with low expression of *Foxn1* and its main targets. Furthermore, male cTECs expressed relatively low levels of genes instrumental in thymocyte expansion (e.g., *Dll4*) and positive selection (*Psmb11* and *Ctsl*). Nevertheless, cTECs were more abundant in males than females. Accumulation of cTECs in males correlated with differential expression of genes regulating cell survival in cTECs and cell differentiation in mTECs. The sexual dimorphism of TECs highlighted here may be mechanistically linked to the well-recognized sex differences in susceptibility to infections and autoimmune diseases.

The immune system of vertebrates shows a major sexual dimorphism. Thus, relative to males, females usually display stronger immune response to vaccination and infection but suffer a higher propensity to many autoimmune diseases^1^,^2^,^3^,^4^. Though we have limited insight into the mechanistic bases for these differences^4^, evidence suggests that they result from both direct and indirect effects of sex steroids on innate and adaptive immune responses. Thus, sex hormones can affect immune cells directly or via modulation of the gut microbiome which in turn influences the immune response^5^,^6^,^7^,^8^,^9^. Sex-based differences in thymus biology have been observed in many studies. Thymic involution, which affects all vertebrates^10^,^11^, accelerates at puberty and progresses at different rates in males and females^12^,^13^,^14^. Also, administration and ablation of androgens respectively accelerates and reverses, albeit transiently, thymic involution^15^,^16^,^17^,^18^,^19^. Nevertheless, it remains unclear to what degree sex steroids’ impact on thymic cellularity may reflect the influence of hormones on thymic epithelial cells (TECs)^3^ vs. other cell types (thymocytes, mesenchymal cells).

Recent studies have shown that sex steroids do affect TECs. In adult mice, cortical and medullary TECs (cTECs and mTECs) have slower proliferation rates in males than females^20^. Furthermore, following acute ablation in a transgenic mouse model, cTECs were found to regenerate in females and castrated (Cx) males, but not in males or androgen-treated females^21^. Finally, it was recently shown that, in TECs, androgens repress transcription of the Notch ligand *Dll4*^22^ which is essential for specification, commitment, and development of thymocytes^23^. Nevertheless, studies in other cell types (e.g., liver, adipose tissue, muscle, and brain) have shown that the effects of sex hormones are pleiotropic and exquisitely cell-type dependent^24^,^25^. Hence, the impact of sex hormones on TECs cannot be inferred from data obtained in other cell types. The main goal of our study was therefore to evaluate at the systems-level, the effect of sex hormones on TEC subsets. To this end, we sequenced the transcriptome of cTECs and mTECs from three groups of 6 month-old mice: males, females and males castrated at four weeks of age. In parallel, we analyzed variations in the size of TEC subsets in those three groups between 1 and 12 months of age. We report that sex hormones have pervasive effects on TEC biology. In particular, adult males accumulate more cTECs than females, but male cTECs display low proliferation rates and show evidence of functional impairment. Furthermore, relative to castrated males, mTECs from males and females showed decreased expression of promiscuously expressed genes.

## Results

### Dynamics of TEC populations as a function of age

We expected that, in addition to divergent effects, sex hormones would have some redundant effects on TECs. Therefore, to get a global picture of these effects, we analyzed not only male and female mice but also Cx male mice. The onset of male puberty in inbred laboratory mice usually occurs between 34 and 38 days after birth^26^. Hence, in order to prevent any exposure to high levels of androgens, castration of male mice was performed before full sexual maturity, at one month of age (see Supplementary Figure S1a for experimental design). Also, knowing that castration induces a transient thymic hypertrophy^16^, Cx males were not studied in the early weeks following surgery; they were analyzed before surgery (1 month-old) and at 3–12 months of age.

**Figure 1:**
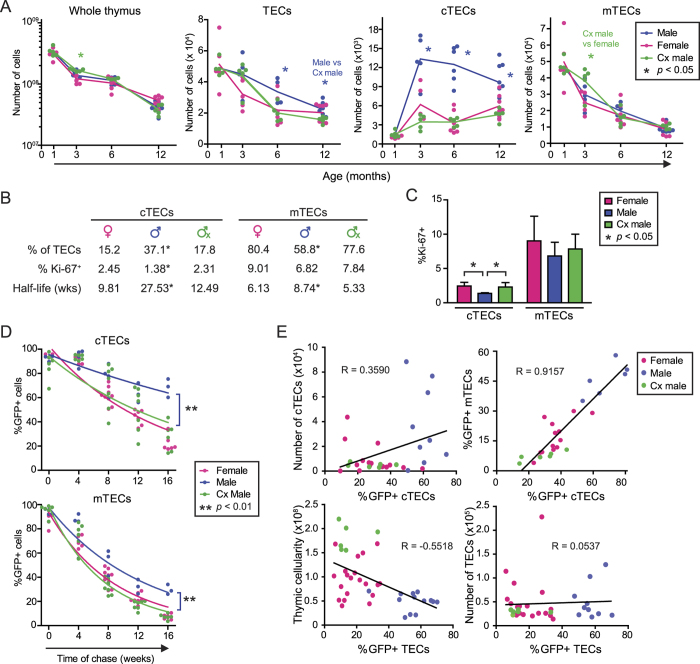
Male mice show an accumulation of cTECs with age that is not due to enhanced cell proliferation. (**a**) Thymic cell populations in mice aged from 1 to 12 months. The gating strategy is depicted in Supplementary Figure S1c. (**b**) Percentage of TEC populations in 6 month-old mice, mean percentage of Ki-67^+^ cells at 4–5 months of age, and half-life (in weeks) of H2B-GFP^+^ TECs calculated using one phase decay nonlinear regression. (**c**) Percentages of Ki-67^+^ TECs in 4 to 5 month-old mice. (**d**) H2B-GFP dilution in adult mice during a chase period of 16 weeks. (**e**) Correlation (Pearson) between different features of TEC populations in 6 month-old mice. Each dot represents onemouse.

Total thymic cellularity, which essentially reflects thymocyte numbers, was similar in the three experimental groups over time (Fig. 1a, left). Only a minimal and transient hypercellularity was observed at three months in Cx males, probably reflective of the ephemeral thymic rebound induced by castration^16^. In adult mice, about 80% of TECs are mTECs and 20% cTECs 20,27. Notably, at 6 and 12 months, males possessed higher numbers of TECs than females and Cx males (Fig. 1a, middle left). This discrepancy was due solely to the greater abundance of cTECs in males, from 3 to 12 months of age (Fig. 1a middle right and 1b). In contrast, the loss of mTECs with age was similar in the three experimental groups (Fig. 1a, right). In order to understand the dramatic differences in cTEC numbers in males relative to other groups, we first analyzed TEC proliferation using two models. In the first model, the percentage of cycling cells was assessed using Ki-67 staining. As previously observed in adult mice^20^, mTECs proliferated more actively than cTECs in all groups (p < 0.05, Fig. 1c). Interestingly, males displayed a significantly lower percentage of Ki-67^+^ cTECs than females and Cx males (Fig. 1c). The mTEC compartment showed a similar tendency which, however, did not reach significance. In the second model, we use the H2B-GFP:rtTA transgenic mouse model in which the extent of cell proliferation can be inferred from H2B-GFP fluorescence intensity (see Supplementary Figure S1b)^20^,^28^. After administration of doxycycline during 6 weeks, TEC populations were analyzed during a chase period of 16 weeks (Fig. 1d). Nonlinear regression analysis of H2B-GFP dilution revealed that cTECs and mTECs in adult mice proliferated less actively in males than in females and Cx males (Fig. 1b,d). The half-life of GFP^+^ TECs was similar in females and Cx males (Fig. 1b). Therefore both methods yielded congruent results showing that cTECs proliferate less actively in males than in females and Cx males. Hence, the greater abundance of cTECs in males cannot be ascribed to a higher proliferation rate. Also, since the level of androgens is the sole feature that differentiates males from both Cx males and females (Supplementary Figure S1a), these results suggest that androgens have an anti-proliferative effect on TECs, and particularly on cTECs.

Next, we evaluated the correlation between TEC proliferation and cellularity after 16 weeks of chase. Surprisingly, there was a modest positive correlation (R = 0.3590) between the percentage of GFP^+^ cTECs and the number of cTECs (Fig. 1e, upper left). This means that thymi in which cTECs proliferate more actively (and therefore have a low percentage of GFP + cells) actually have less cTECs. As a corollary, there must be a greater loss of cTECs in females and Cx males than in males. Two non-mutually exclusive mechanisms may be responsible for cTEC loss: cTEC apoptosis vs. differentiation of cTEC-phenotype cells into mTEC-phenotype cells. The latter possibility must not be discarded since the cTEC compartment (EpCAM^+^Ly51^+^UEA1^–^) contains bipotent TEC progenitors that can ultimately adopt a cTEC or an mTEC fate^29^,^30^. Besides, proliferation of cTECs was highly correlated with mTEC proliferation (R = 0.9157) (Fig. 1e, upper right). Furthermore, global TEC proliferation showed a significant correlation (R = 0.5518) with overall thymic cellularity (i.e., thymocyte numbers; Fig. 1e, lower left) but not with TEC numbers (Fig. 1e, lower right). The facts that cTEC proliferation was positively correlated with mTEC proliferation and thymocyte numbers suggests that cell proliferation in these three cell compartments may be co-regulated.

### The impact of sex on the transcriptome of TEC subsets–An overview

**Figure 2:**
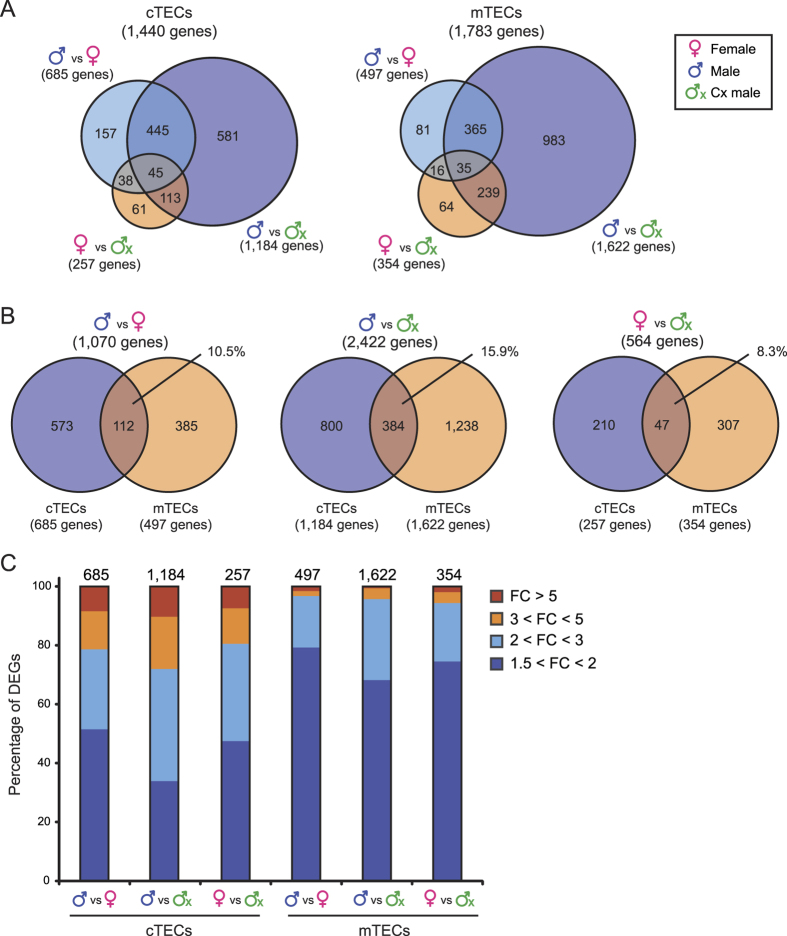
Transcriptomic signatures of cTECs and mTECs in 6 month-old male, female and Cx male mice. (**a**) Venn diagram representation of DEGs in cTECs and mTECs from the three experimental groups. (**b**) Overlap between DEGs in cTECs and mTECs. (**c**) Percentages of DEGs for selected thresholds of mRNA expression fold-change (FC). Numbers of DEGs are indicated above bars.

In order to assess the extent and molecular bases of sexual dimorphism in adult TECs, we performed RNA sequencing on cTECs and mTECs from 6 month-old mice from our three experimental groups (see gating strategy in Supplementary Figure S1c). DEGs were identified with the DESeq package^31^ using the following criteria: adjusted p value < 0.1, fold change ≥1.5 and minimal mean expression of 1 RPKM. A total of 1,440 and 1,783 DEGs were found in the three experimental groups in cTECs and mTECs, respectively (Fig. 2a). As observed in other tissues, sex-related differences in gene expression were generally modest, being <3-fold in most cases (Fig. 2c). Males and Cx males showed the greatest number of DEGs, with 1,184 (82%) and 1,622 (91%) DEGs in cTECs and mTECs, respectively. This result was striking since the sole physiological difference between these two groups is the level of androgens (Supplementary Figure S1a). Together with the relatively low number of DEGs in females vs. Cx males (Fig. 2a), these data indicate that androgens have a greater impact on TEC transcriptome than female sex hormones or the type of sex chromosomes. Previous studies on other types of somatic cells have shown that sexual dimorphism in gene expression is highly tissue-specific^24^,^25^. This was vividly illustrated by the fact that in various pairwise comparisons, we found that only 8 to 16% of DEGs were common to cTECs and mTECs (Fig. 2b).

### Androgens inhibit cell death and cell differentiation in TECs

**Figure 3:**
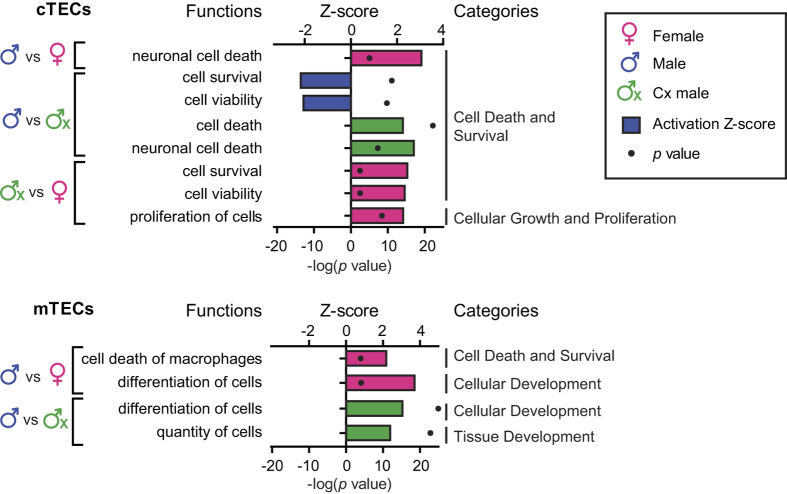
IPA analysis of DEGs in cTECs and mTECs. IPA analysis of DEGs predicts decreased cell death and cell differentiation in males. Activation Z-scores are depicted with bars, whereas p values are shown with black dots. The color of bars shows in which group a given process is activated (e.g., blue = males). All functions represented on the graph were significantly enriched (p < 0.05).

To better understand of the impact of sex hormones on TEC function, we performed a downstream effect analysis on our lists of DEGs using the Ingenuity Pathway Analysis software (IPA, Ingenuity® Systems, www.ingenuity.com). We used two metrics to identify the most important downstream effects of these DEGs: activation Z-score and p-value. Positive and negative Z-scores indicate increased and decreased functional activity, respectively. The p-value, calculated with the Fischer’s exact test, indicates the likelihood that the association between a set of DEGs and a biological function is significant. The complete list of biological processes regulated by DEGs can be found in Supplementary Figures S2 and S3. Of particular relevance in view of differences in TEC subsets depicted in Fig. 1, the following functional categories were highlighted as being differentially activated in the three experimental groups: cell death and survival and cell differentiation (Fig. 3). More specifically, IPA analysis predicted that, relative to the two other groups, male TECs would show i) less cell death (cTECs and mTECs) and ii) less cell differentiation (mTECs). Superior cTEC survival coupled to lessened differentiation of cTEC-phenotype progenitors into mTECs offer a plausible explanation for the higher numbers of cTECs observed in males (Fig. 1a).

**Figure 4:**
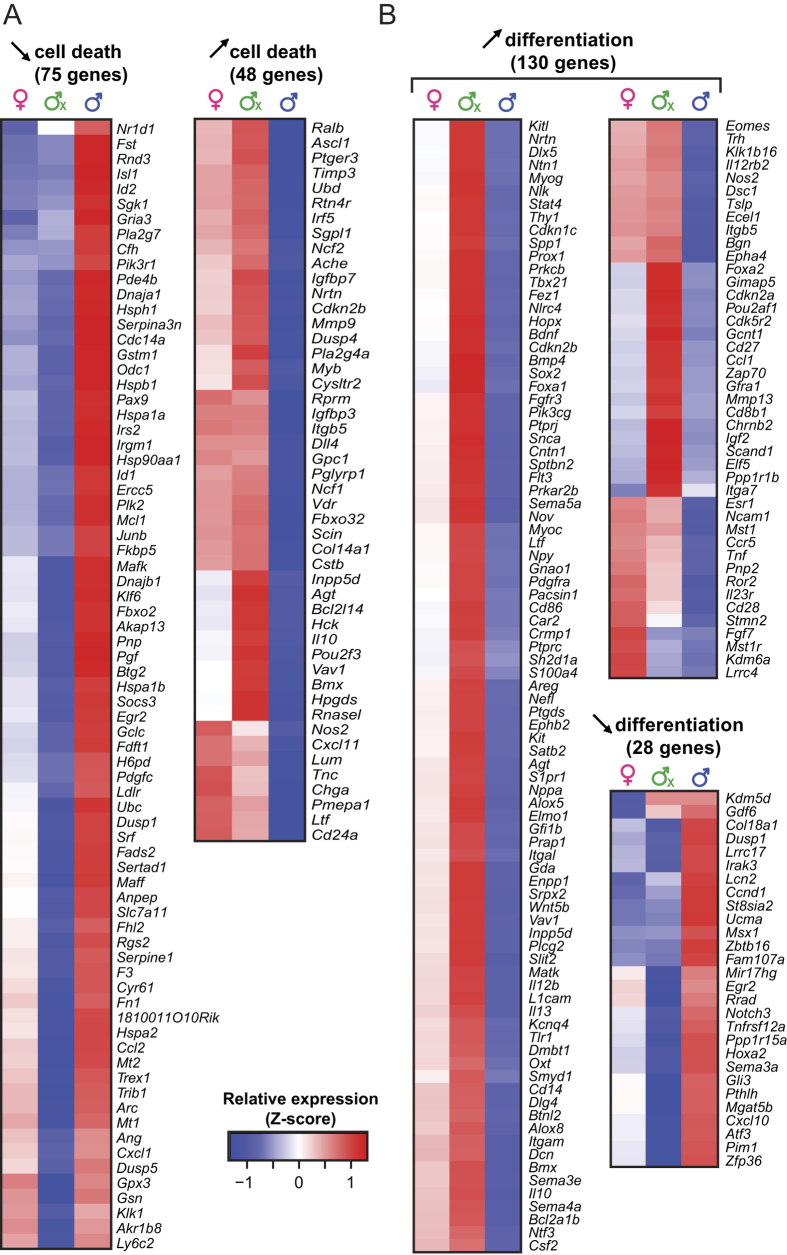
Relative expression of genes contributing to the inhibition of cell death and cell differentiation in male TECs. (**a**) Relative expression of genes that affect cell death in cTECs. (**b**) Relative expression of genes that affect cell differentiation in mTECs. Relative gene expression is depicted as a Z-score, calculated separately for cTECs and mTECs. Red corresponds to higher expression, whereas blue corresponds to lower expression.

We then sought to gain further mechanistic insights into processes regulated by sex hormones by analyzing relevant DEGs, i.e. genes whose differential expression was consistent with the predicted activation status in the three experimental groups (Fig. 4). Genes were separated into activators or inhibitors of cell death or cell differentiation. The dataset related to cell death included DEGs associated with the following functional categories: cell death and survival, cellular growth and proliferation, and tissue development. Notably, *Pax9* and *Sgpl1*, which are known to regulate TEC survival and apoptosis^32^,^33^, were among DEGs linked to inhibition of cell death in male TECs (Fig. 4a). Furthermore, many genes known to regulate survival of different epithelial cell types are present in that list, including *Sgk1*, *Id1*, *Vdr*, *Bcl2l14*, *Dusp1*, *Bmx* and *Igfbp3* (mammary gland), *Pgf*, *Irf5*, *Pla2g4a* and *Pmepa1* (intestine), and *Hspa1a* (ovary)^34^,^35^,^36^. Finally, several members of key pathways regulating cell survival/death were among sexually dimorphic genes: heat shock proteins (*Hspa1a*, *Hspa1b*, *Hspa2*, *Hspb1*) and members of the p53 (*Rnd3*, *Plk2*, *Sgpl1*, *Igfbp3*, *Pmepa1*), Pi3k/Akt (*Pik3r1*, *Pdgfc*, *Fn1*) and Fas/caspases pathways (*Mcl1*, *Hspa1b*, *Fn1*, *Bcl2l14*, *Lum*).

On the other hand, many genes which are instrumental in differentiation of ectoderm-and endoderm-derived epithelia were differentially expressed and may therefore contribute to the inhibition of cell differentiation in male mTECs: *Elf5*, *Oxt*, *Dmbt1*, *Areg, Spp1* (mammary gland), *Kitl*, *Alox8*, *Dsc1*, *Atf3* (skin), *Notch3*, *Mir17hg* and *Foxa2* (lung epithelial cells), *Ccnd1*, *Cdkn2a* and *Dmbt1* (colon)^37^,^38^,^39^,^40^. In conclusion, IPA analysis provides a molecular framework that can explain why cell death and cell differentiation are inhibited in male TECs relative to female and Cx male TECs.

### Androgens impact on genes involved in thymopoiesis

**Figure 5:**
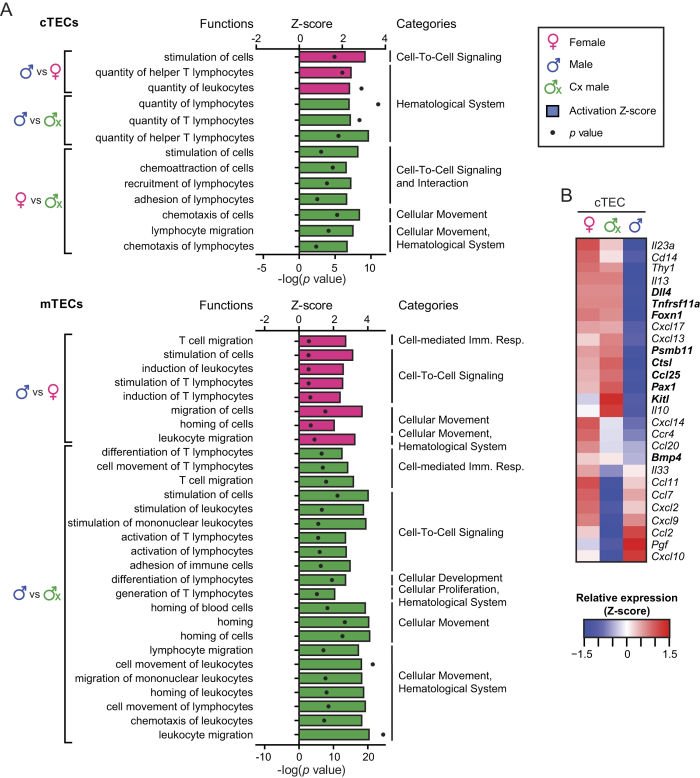
Several TEC genes involved in thymopoiesis are downregulated in male TECs. (**a**) IPA analysis of DEGs predicts differential activation of biological functions related to homing, stimulation of cells, chemotaxis and production of T lymphocytes. Activation Z-score is depicted with bars, whereas p values are shown with black dots. The color of bars shows in which group a given process is activated (e.g., green = Cx males). All functions represented on the graph are significantly enriched (p < 0.05). (**b**) Heatmaps of relative cTEC expression of IPA-discovered genes that may affect thymopoiesis. Genes that have well-characterized functions in the thymus are highlighted in bold. The gene expression is depicted as a Z-score, calculated separately for each cell type. Red corresponds to higher expression, whereas blue corresponds to lower expression.

IPA analysis of DEGs in the three experimental groups predicted that several functions linked to T-cell production were inhibited in male TECs. These functions included stimulation, homing and chemoattraction of lymphoid cells (Fig. 5a). DEGs involved in these functional categories included several cytokines, transcription factors and costimulatory molecules of prime relevance in thymopoiesis (Fig. 5b). *Foxn1*, the key regulator of thymus development and maintenance^41^,^42^, was downregulated by ~2 fold in cTECs from males relative to females and Cx males. In accordance with this, expression of *Foxn1* targets *Ccl25*, *Dll4, Pax1* and *Kitl*^43^,^44^ was also lower in male cTECs. Other genes having a crucial role in thymocyte development^45^,^46^,^47^ were also downregulated in male cTECs: i) *Psmb11* (AKA *β5t*), a cTEC-specific proteasome subunit required for positive selection of CD8 thymocytes^48^, ii) *Ctsl*, a peptidase instrumental in the positive selection of CD4 thymocytes^49^ and iii) *Tnfrsf11a* (AKA RANK), the TNF family receptor necessary for mTEC maturation^50^. Overall, these data suggest that androgens have a negative impact on TEC-mediated processes that are central to normal thymopoiesis.

### Cx males show increased promiscuous gene expression in mTECs

**Figure 6:**
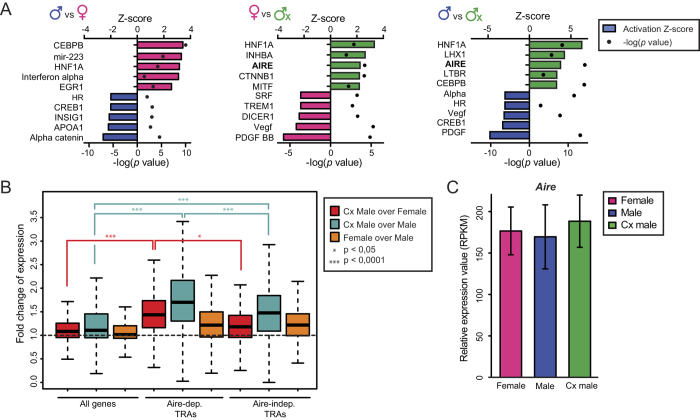
Cx males show higher expression of TRAs. (**a**) Ten most activated upstream regulators predicted by IPA analysis of mTECs DEGs in female vs male (left), in Cx male vs female (center) and in Cx male vs male (right, all predicted activators shown are significant p < 0.05). The color of bars shows in which group a given upstream regulator is activated (e.g., blue = males). (**b**) Fold-difference in expression of all genes, *Aire*-dependent TRAs and *Aire*-independent TRAs in mTECs. The gene expression ratio (log2 RPKM) of Cx male over female is depicted in red, of Cx male over male in turquoise and of female over male in orange. (**c**) Relative expression of *Aire* in mTECs (RPKM).

Induction of so-called “central tolerance” in the thymic medulla depends on ectopic expression of proteins otherwise restricted to differentiated organs in the periphery^51^. Collectively, mTECs express almost all protein-coding genes^52^,^53^ and can therefore induce tolerance to a wide array of tissue-restricted antigens (TRAs). Promiscuous gene expression by mTECs is therefore essential to induce tolerance to “extrathymic proteins”, and is regulated in part by the autoimmune regulator (AIRE). According to IPA analysis, several upstream regulators were differentially activated in mTECs from our three experimental groups (Fig. 6a). One of the top regulators was *Aire*, which was activated in mTECs from Cx males compared to males and females (Fig. 6a). This led us to evaluate expression of TRAs in mTECs from our three groups. We adopted the definition stipulating that TRA-coding genes are those that are tissue-enriched (*i.e.* expressed at relatively high levels) in at most five tissues^54^. Our definition of AIRE-dependent and –independent TRAs was based on RNA-seq analysis of mTECs from wild-type and *Aire*^*−/−*^ littermates^55^. We found that *Aire*-dependent TRAs were significantly upregulated in Cx males compared to females (red) and males (turquoise), whereas males and females showed no difference (orange, Fig. 6b). Furthermore, Cx males expressed higher levels of *Aire*-independent TRAs than males, although the difference was less than for *Aire*-dependent TRAs (Fig. 6b). However, *Aire* expression was similar in the three experimental groups (Fig. 6c). These data indicate that the differential abundance of TRAs was not due to differential expression of *Aire*. In addition, they argue against the possibility that Cx males might have a higher proportion of mature mTECs than other groups, since mTEC maturity correlates with *Aire* expression. We therefore conclude that sex hormones directly or indirectly repress the promiscuous gene expression of *Aire*-dependent and –independent TRAs, without affecting the expression of *Aire* itself.

## Discussion

The present work shows that sex hormones have pervasive effects on the transcriptome of TECs: the number of DEGs was 1,440 in cTECs and 1,783 in mTECs (Fig. 2a). Studies on adipocytes as well as brain, liver and muscle cells revealed that sex-related changes in gene expression were highly tissue-specific^24^,^25^. It is remarkable that the same holds true in cells that are as closely related, ontogenetically, as cTECs and mTECs. Indeed, the overlap between cTEC DEGs and mTEC DEGs in various pairwise comparisons was only about 12% (Fig. 2b). This means that the sex differences are exquisitely context dependent.

Sexual dimorphism was particularly conspicuous in cTECs where it was mainly regulated by the levels of testicular androgens. Androgens induced an accumulation of cTECs that were hypoproliferative and presented features suggestive of functional impairment. The low proliferation rate of male TECs can be explained by a low expression of *Foxn1* and its main targets, which are required for both fetal and adult thymopoiesis^41^. In adult mice, minor variations in the expression or function of *Foxn1* directly impact on thymic cellularity^42^,^56^. Interestingly, thymic cellularity (i.e., number of thymocytes) correlated with TEC proliferation (higher in females) (Fig. 1e) but not with cTEC or total TEC numbers (higher in males) (Fig. 1a,e). This observation is consistent with the fact that thymic output in human adults (number of signal-joint T cell receptor excision circles/T cell) is higher in females than males^13^. In addition, numerous genes that are instrumental in thymocyte development were repressed in male cTECs (Fig. 5). These DEGs included genes driving thymocyte expansion (e.g., *Dll4*) and positive selection (*Psmb11* and *Ctsl*) and whose repression can certainly impinge on thymopoietic activity^22^.

But if cTECs proliferate more in females than males, why are they less abundant in females? Our IPA analyses suggest that cTEC accumulation in males results from enhanced survival coupled to impairment of cell differentiation (Fig. 4). Given the large numbers of relevant DEGs, direct evaluation of the role of individual genes would require numerous gain-and loss-of-function experiments. Finally, is there any rationale for the superior proliferative potential of female over male cTECs? Our favorite hypothesis is that evolution has favored high cTEC renewal potential in females because of the constraints imposed by pregnancy. Pregnancy causes a major, though transient, TEC-dependent thymic involution which is instrumental in enhancing reproductive fitness^17^,^57^. This process is progesterone-dependent, and transplantation of a progesterone receptor null thymus in wild-type female mice impairs fertility. Relative to recipients of a wild-type thymus, mice grafted with a progesterone receptor null thymus have smaller litters and higher numbers of resorbing embryos^57^. We therefore speculate that a high cTEC renewal potential allows females to withstand pregnancy-associated thymic involution and regain adequate thymic function rapidly in the postpartum period.

Unexpectedly, we found that both female and male sex hormones repressed promiscuous gene expression in mTECs. Both AIRE-dependent and –independent TRAs were expressed at higher levels in Cx males than in females and males. Hence, while sex hormones did not affect the expression of *Aire* per se, they must impinge on the activity of AIRE cofactors or unidentified regulator(s) of promiscuous gene expression in mTECs. Is this effect biologically significant? We speculate that, at least for the *Aire*-dependent TRAs, lower TRA expression in adult is probably not biologically important under physiological conditions. Indeed, Aire-controlled mechanisms of central tolerance are largely dispensable in the adult^58^. However, we hypothesize that the effect of sex hormones on TRA expression may be highly relevant in the context of allogeneic hematopoietic cell transplantation (AHCT) in adults. Indeed, following AHCT, donor hematopoietic progenitors must undergo a second round of ontogeny in the recipient’s thymus, in the presence of sex hormones if the recipient is an adult. During this period, the occurrence of graft-versus-host disease in many recipients leads to loss of AIRE^+^ mTECs and thereby to generation of autoreactive T cells^59^. By extension, our data would suggest that sex hormones might have a negative impact on the establishment of graft-host tolerance and might explain why post-AHCT autoimmunity [i.e., chronic graft-versus-host disease^59^] is much more common in adults than children^60^. If this were the case, pharmacological antagonists of sex hormones could mitigate the risk of chronic graft-versus-host disease, which remains the unrelenting nemesis of patients and physicians involved in AHCT^60^,^61^.

The sexual dimorphism of TECs highlighted here may be mechanistically linked to the well-recognized sex differences in susceptibility to infections and tissue-specific and systemic autoimmune diseases^4^. While these complex issues may be tackled from different perspectives, our next objective will be to investigate whether the sexual dimorphism of cTECs impacts on the diversity of the T-cell repertoire.

## Methods

### Mice

C57BL/6 mice, B6.Cg-*Gt(ROSA)26Sor*^tm1(rtTA*M2)Jae^/J and STOCK Tg(tetO-HIST1H2BJ/GFP)47Efu/J mice purchased from The Jackson Laboratory (Bar Harbor) were bred and housed under specific-pathogen-free conditions in sterile ventilated racks at the Institute for Research in Immunology and Cancer. For H2B-GFP pulse-chase experiments, doxycyline was incorporated in food (2 g/kg) (Hartlan Laboratories) or in drinking water (2 mg/ml of doxycycline supplemented with 5% sucrose) (Sigma Aldrich)^20^. The pulse period lasted for six weeks and was initiated at 4–6 weeks of age. All procedures were in accordance with the Canadian Council on Animal Care guidelines and approved by the Comité de Déontologie et Expérimentation Animale de l’Université de Montréal.

### Castration

Four week-old male mice were anesthetized and a small incision in the scrotal region was made to expose the testes. Testes were tied off with suture thread and removed.

### Flow cytometry analysis and sorting

Enrichment of thymic stromal cells was performed as previously described^52^,^62^. Thymic stromal cells were stained with biotinylated Ulex Europaeus Lectin 1 (UEA1; Vector Laboratories) and PE-Cy7 or PE-TexasRed conjugated streptavidin (BD Biosciences) and the following antibodies: i) AlexaFluor-700 anti-CD45 from BD Biosciences, ii) AlexaFluor-647 anti-Ly51 and APC-Cy7 anti-EpCAM from BioLegend. Staining with 7-AAD and PE conjugated anti-Ki-67 (BD Biosciences) was used to assess cell viability and the proportion of cycling cells. TECs were selected as CD45^−^EpCAM^+^, and mTECs and cTECs were defined as UEA-1^+^Ly51^−^ and UEA-1^−^Ly51^+^, respectively. Intranuclear staining of Ki-67 was performed using the Foxp3/Transcription Factor Staining Buffer Set (Affymetrix eBiosciences). For flow cytometry analyses, each group contained 4–8 mice per time point. Live cTECs and mTECs were sorted on a three laser FACSAria or analyzed on a three laser LSR II using FACSDiva (BD Biosciences) and FlowJo VX.0.7(FlowJo Enterprise) softwares.

### RNA sequencing experiments

We analyzed the transcriptome of six populations of TECs: cTECs and mTECs from males, females and Cx males. We obtained three biological replicates from each TEC population, except for Cx male mTECs where we had 2 biological replicates. Hence, in toto, we analyzed 17 biological replicates (Supplementary Table S1). Each biological replicate contained pooled FACs-sorted cTECs or mTECS from four to six mice. Total RNA was isolated using Trizol^TM^ as recommended by the manufacturer (Invitrogen), and then further purified using RNeasy Micro columns (Qiagen). Sample quality was assessed using Bioanalyzer RNA Pico chips (Agilent). Transcriptome libraries were made using the TruSeq RNA Sample Prep Kit (v2) (Illumina) following the manufacturer’s protocols. Library generation was then assessed using a Bioanalyzer platform (Agilent) and Illumina MiSeq-QC run. Then, sequencing was done using an Illumina HiSeq2000 using TruSeq SBS v3 chemistry at the Institute for Research in Immunology and Cancer’s Genomics Platform. Cluster density was targeted at around 800 k clusters/mm^2^. Data was mapped to the Mus musculus (mm10) reference genome using the ELANDv2 alignment tool from the CASAVA 1.8.2 software (Illumina). RNA-seq data have been deposited in GEO archives (http://www.ncbi.nlm.nih.gov/geo/) under accession number GSE66873 (http://www.ncbi.nlm.nih.gov/geo/query/acc.cgi?acc=GSE66873) and are displayed in Supplementary Table S1. Analyses of RNA sequencing data were performed using the publicly available statistical software package “R” (http://www.r-project.org/). Differentially expressed genes (DEGs) were determined with the DESeq package from Bioconductor (http://bioconductor.org/), using thresholds of adjusted p-value of 0.1 and a minimal fold change of 1.5. To remove genes that were lowly expressed in our analysis, we further filtered the DEGs to only keep genes that had at least one sample with a relative expression higher than 1 read per kilobase of exon model per million reads mapped (RPKM). Enrichment of biological functions and predicted upstream regulators were assessed using the IPA software (Ingenuity Systems, http://www.ingenuity.com).

### Statistical analyses

Unless stated otherwise, results are expressed as means ± SD, statistical significance was tested using Student unpaired two-tailed t test, and differences with a *p* value < 0.05 were considered significant. Significant differences in the percentages of Ki-67+ cells were assessed using a one-way ANOVA and a Newman-Keuls post-hoc test. The half-life of GFP^+^ cells were calculated using a one-phase decay nonlinear regression analysis and significance was assessed using an extra sum-of-squares F test. Correlations were calculated using Pearson’s correlation test. All statistical analyses were performed with GraphPad Prism software V5.01.

## Additional Information

**How to cite this article**: Dumont-Lagacé, M. *et al.* Sex hormones have pervasive effects on thymic epithelial cells. *Sci. Rep.* **5**, 12895; doi: 10.1038/srep12895 (2015).

## Supplementary Material

Supplementary Information

Supplementary Dataset 1
