# Supplementary material for: Sex hormones have pervasive effects on thymic epithelial cells
Source: Sci Rep. 2015 Aug 7;5:12895. doi: 10.1038/srep12895 (PMC4528223; doi:10.1038/srep12895)
Supplement: Supplementary Information [file srep12895-s1.pdf]

Sex hormones have pervasive effects on thymic epithelial cells

Subjects: Gene expression, Hormone receptor, Immunology, Physiology, Systems biology

Maude Dumont-Lagacé, Charles St-Pierre and Claude Perreault

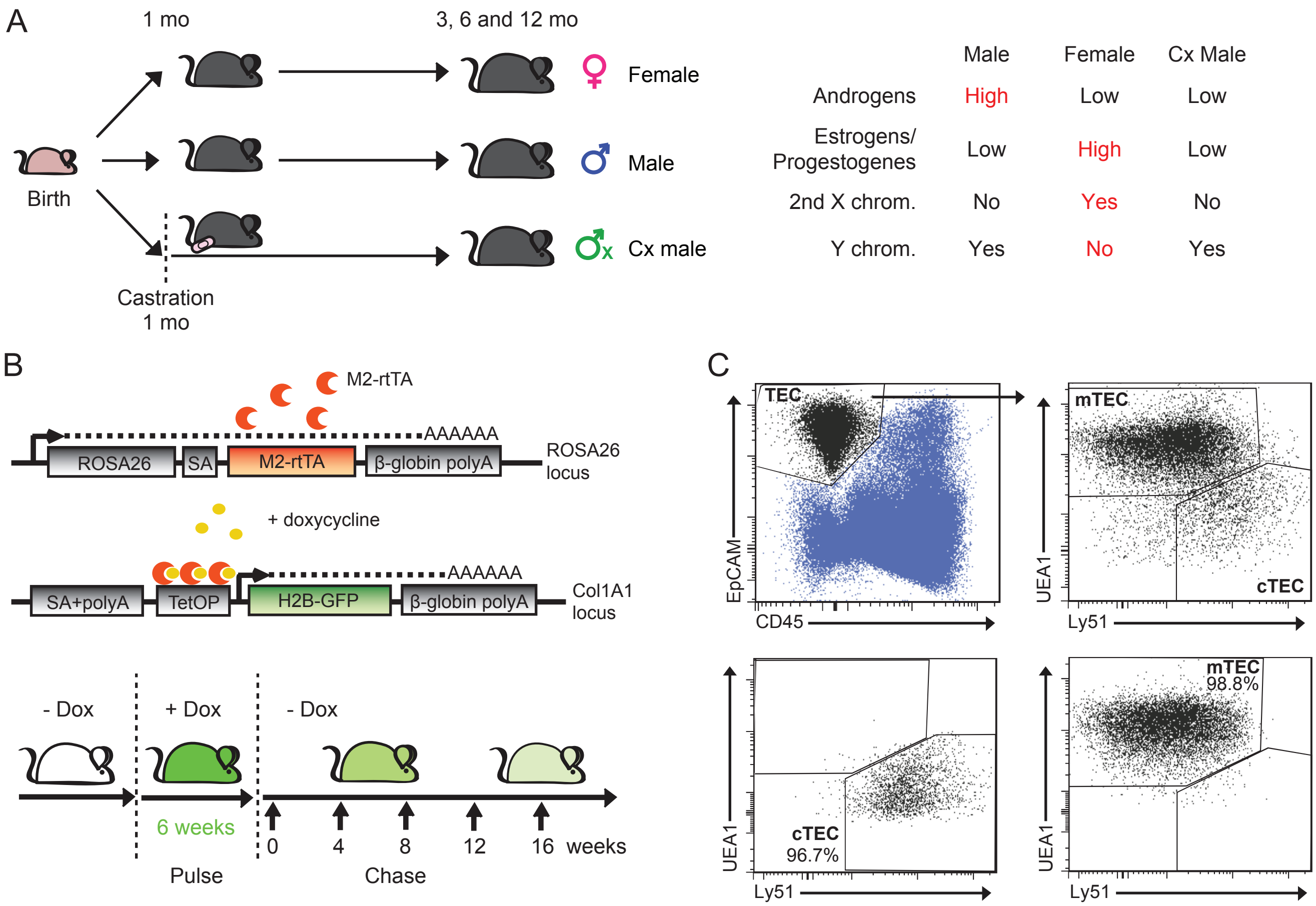

Supplementary Figure S1: Experimental design for phenotypic and transcriptomic analyses of male, female and Cx male TECs. (A) Castration was performed at 1 month of age, and mice were analysed at 1, 3, 6 and 12 months of age. The table on the right displays the physiological differences that can directly account for differences between the experimental groups. Features unique to a group are in red, whereas features that can be found in two groups are in black. (B) The H2B-GFP:rtTA mouse model. Administration of doxycycline activates the M2-rtTA transactivator and allows the expression of H2B-GFP during the pulse period. The H2B-GFP will be incorporated in the nucleus during cell division in the pulse period (6 weeks) and segregates equally in the daugther cells during cell division during the chase period (16 weeks). (C) Gating strategy to identify and sort cTECs and mTECs (TECs: EpCAM+CD45-, cTECs: Ly51+UEA1- and mTECs: Ly51-UEA1+). Below: post-sort analysis of sorted cTECs and mTECs. The figure was drawn by Maude Dumont-Lagacé.

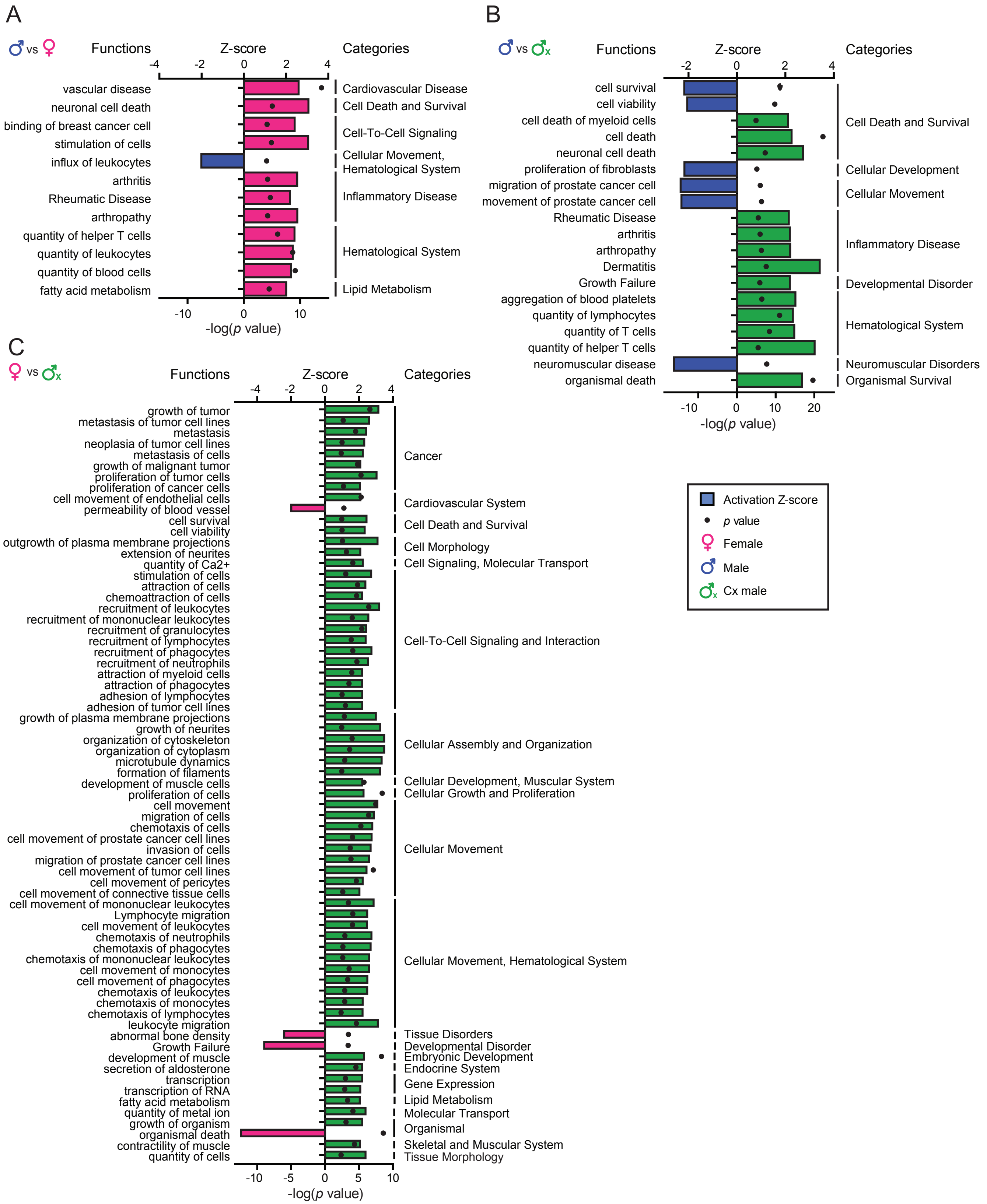

Supplementary Figure S2: Differentially activated functions in cTECs according to IPA analysis. (A) Analysis of DEGs between males and females, (B) between males and Cx males and (C) between females and Cx males. Activation Z-score is depicted in bars, whereas *p* values are depicted with black dots. The color of bars shows in which group a given process is activated (e.g., blue = males). All functions represented are significant ($p < 0.05$).

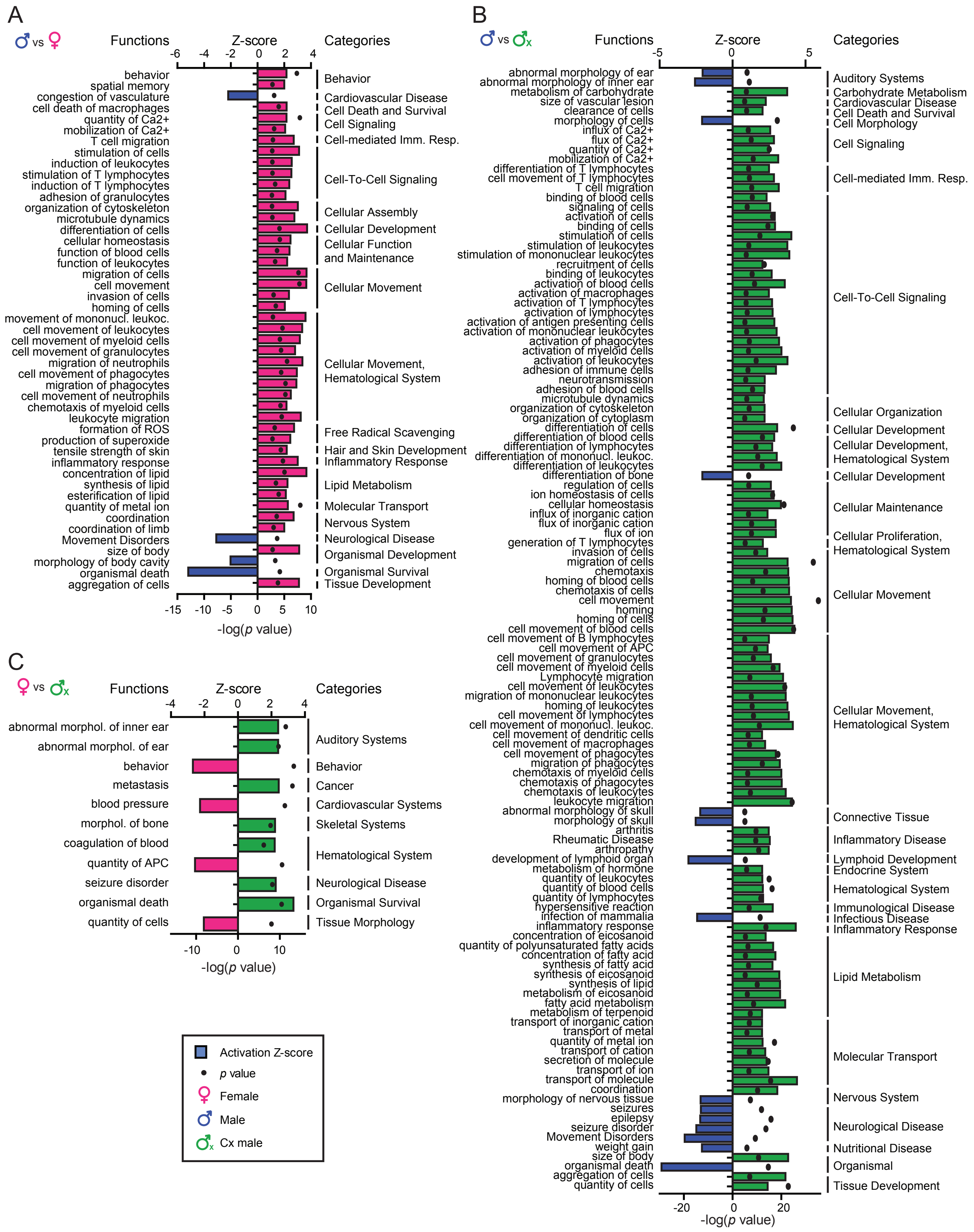

Supplementary Figure S3: Differentially activated functions in mTECs according to IPA analysis. (A) Analysis of DEGs between males and females, (B) between males and Cx males and (C) between females and Cx males. Activation Z-score is depicted in bars, whereas *p* values are depicted with black dots. The color of bars shows in which group a given process is activated (e.g., blue = males). All functions represented are significant ($p < 0.05$).
